# The efficacy of muscle energy techniques in symptomatic and asymptomatic subjects: a systematic review

**DOI:** 10.1186/s12998-019-0258-7

**Published:** 2019-08-27

**Authors:** Ewan Thomas, Antonio Rosario Cavallaro, Diba Mani, Antonino Bianco, Antonio Palma

**Affiliations:** 1 Sport and Exercise Sciences Research Unit, University of Palermo, Via Giovanni Pascoli 6, 90144 Palermo, Italy; 2 International Academy of Osteopathic Medicine, AISeRCO, Palermo, Italy; 3 Department of Integrative Physiology, University of Colorado, Boulder, CO USA; 4 Department of Applied Physiology and Kinesiology, University of Florida, Gainesville, FL USA

**Keywords:** Manipulative therapies, Pain, Range of motion

## Abstract

**Background:**

Muscle energy techniques are applied to reduce pain and increase range of motion. These are applied to a variety of pathological conditions and on asymptomatic subjects. There is however limited knowledge on their effectiveness and which protocol may be the most beneficial.

**Objective:**

The aim of this review is to determine the efficacy of muscle energy techniques (MET) in symptomatic and asymptomatic subjects.

**Design:**

Systematic Review.

**Methods:**

A literature search was performed using the following database: Cochrane Library, MEDLINE, NLM Pubmed and ScienceDirect. Studies regarding MET in asymptomatic and symptomatic patients were considered for investigation. The main outcomes took into account range of motion, chronic and acute pain and trigger points. Two trained investigators independently screened eligible studies according to the eligibility criteria, extracted data and assessed risk of bias. Randomized control trials (RCT’s) were analyzed for quality using the PEDro scale.

**Results:**

A total of 26 studies were considered eligible and included in the quantitative synthesis: 14 regarding symptomatic patients and 12 regarding asymptomatic subjects. Quality assessment of the studies through the PEDro scale observed a “moderate to high” quality of the included records.

**Conclusions:**

MET are an effective treatment for reducing chronic and acute pain of the lower back. MET are also effective in treating chronic neck pain and chronic lateral epicondylitis. MET can be applied to increase range of motion of a joint when a functional limitation is present. Other techniques seem to be more appropriate compared to MET for trigger points.

## Introduction

Muscle energy techniques (MET) were originally developed by two osteopathic physicians, Fred Mitchell, Sr. and Fred Mitchell, Jr., to treat soft tissue, mobilize joints, stretch tight muscles and fascia, reduce pain and to improve circulation and lymphatic drainage [1, 2]. MET are defined as a manual treatment in which a patient produces a contraction in a precisely controlled position and direction against a counterforce applied by a manual therapist [3]. It could be advocated that MET are similar to proprioceptive neuromuscular facilitation stretching (PNF) [4]; however, the execution of MET is usually performed with lower forces compared to those of PNF in order to recruit tonic muscle fibers that are associated with tonic motor units which require lower action potentials in order to be recruited than phasic muscle fibers. These latter are activated during PNF and typically occur at forces greater than 25% of the person’s maximal force [5]. Another difference between MET and PNF is that the contraction during MET is performed at the initial barrier of tissue resistance, rather than at the end of the range of motion (ROM) of a joint [6].

Many studies have applied MET in patients with acute and chronic low back pain (LBP) [7–10], latent trigger points [11, 12], cervical pain [13, 14] and other musculoskeletal dysfunctions [15–17]. MET have been also used in asymptomatic subjects in order to increase mobility [18–20]. There is varying evidence that when a joint has a functional limitation, the application of a MET can increase its ROM [15]. In particular, Lenehan et al. [21] applied MET on the thoracic spine in asymptomatic subjects presenting with a restricted and a non-restricted side. MET were able to increase mobility only on the restricted side.

MET protocols developed differ in paradigms, such as in the number of repetitions, strength of contraction, duration of stretch phase and duration of relaxation phase [11, 15, 17, 19]. Two of the most prominent MET typologies of application are those advocated by Greenman [3] and Chaitow [5]. The first involves the application of three to five repetitions with a relaxation phase long enough to reduce the tension of the targeted tissue (usually 5 to 7 s), whereas the second involves the application of four repetitions with a relaxation/stretch phase of 30 to 60 s after each contraction [19]. Other authors have also used similar MET applications [21–23], although a consensus of which protocol could be more effective still needs to be investigated [1]. Aside from the form of application, the main physiological mechanism proposed for MET [5] involves two general principles: (1) post-isometric relaxation [24], which causes a reduction in the tone of a muscle following an isometric contraction and (2) reciprocal inhibition [25], which involves the reduction in tone of the antagonist muscle following the isometric contraction of the agonist muscle through inhibition of the alpha motor neuron. Post-isometric relaxation is the most frequently applied approach, while reciprocal inhibition is used when a tissue has severe limitations or has become fibrotic, as a treatment modality associated to post-isometric relaxation [5]. Although many texts advocate these as the principal mechanisms responsible for muscle relaxation, it has been seen in studies analyzing joint extensibility that including a preisometric contraction, does not alter resting EMG activity, notwithstanding increased ROM [26]. In addition, another study evaluating motor neuron activity, has shown increases in EMG activity also following concentric and eccentric contractions. A greater increase was seen in the first case while such increase was less pronounced in the latter [27]. The exact mechanism for MET-induced pain relief is still unknown, although it has been proposed that MET act on joint proprioceptors and mechanoreceptors that will result in an effect on descending pathways, changing the motor programming of the target joint [1, 22, 28, 29]. It has also been advocated that the reduction of pain and increased mobility are due to changes in the viscoelastic properties of the soft tissue followed by the application of the technique; the mechanism for increased flexibility has been attributed to an increase in stretch tolerance [4, 22]. Very few studies investigating the effects of the different typologies and efficacies of MET have been performed; therefore, the aim of this review is to understand the efficacy of MET specifically on pain and joint range of motion and to understand the differences between the different MET protocols in symptomatic and asymptomatic subjects.

## Materials and methods

This systematic review aims to determine if muscle energy technique may be effective on pain or may increase range of motion of a joint and if such techniques are applied using different protocols. The PRISMA (Preferred Reporting Items for Systematic Reviews and Meta-Analyses) statement was adopted [30]. Studies that include randomized control trials (RCT) analyzing symptomatic patients with various conditions and studies applied on asymptomatic subjects with range of motion limitations were reviewed. All the manuscripts that were RCT’s were assessed for methodological quality using the Physiotherapy Evidence Database (PEDro) scale.

### Inclusion and exclusion criteria

Studies that met the following criteria were included or excluded in this systematic review.

### Study designs

This review includes studies that have applied MET in both asymptomatic subjects and symptomatic patients. In particular, RCT’s analyzing patients who have been treated for their condition using MET and which include a control group, were selected for the symptomatic population. Studies that do not have a control group or a comparator were not considered for inclusion. RCT’s, pretest-posttest and quasi-experimental studies analyzing the effect of MET were also included for the asymptomatic subjects. Reviews, systematic reviews and meta-analysis were not considered.

### Participants

All the analyzed participants were adults to whom a MET had been applied. Children were not considered for analysis.

### Interventions

The interventions that applied MET in both asymptomatic and symptomatic participants described in the eligible studies will be included in this review. For the asymptomatic participants the interventions aimed to increase range of motion and reduce tenderness when present. For symptomatic participants the interventions aimed to reduce either acute or chronic pain. According to the nature of the variables of each study, pain was measured through different means.

### Comparators

Comparators were control groups for the symptomatic population and control groups or other interventions for the asymptomatic participants.

### Outcomes

The primary outcomes were changes in range of motion (ROM) and pressure pain thresholds (PPT) after the MET application in the asymptomatic participants and change in the pain and disability indexes after the MET application in the symptomatic population. PPT, the pain indexes and disability indexes, for this review varied across studies. Another outcome that was analyzed across the review for both asymptomatic and symptomatic participants was the MET protocol used. Each protocol will be considered in terms of number of contractions, seconds which the contraction is applied to the targeted joint, the contraction force applied, the relaxation phase and the stretch, if applicable.

### Search strategy

The literature search was conducted on the following databases: Cochrane Library, MEDLINE, NLM Pubmed, and ScienceDirect. Due to the extensive nature and variety of topics covered by the search, there was no limit to the search period, however the search ended on June 2018. The search strategy was conducted using the following keywords: muscle, energy, technique and MET. These keywords were used as follows: muscle AND energy AND technique OR MET.

After the initial title screening, inclusion and exclusion criteria above described were applied for abstract selection. The inclusion criteria were: (1) studies had to be peer-reviewed, (2) studies had to be performed on adult subjects, (3) studies had to clearly specify the MET procedure used, (4) studies had to report an objective outcome and (5) studies concerning symptomatic patients had to be RCT’s. The exclusion criteria were: (1) Non-English manuscripts, (2) studies performed on children, (3) non-RCT’s studies, (4) studies that did not specify a MET procedure. Following the inclusion of the selected manuscripts, these were divided into two groups: those applying MET on asymptomatic participants and those applying MET on symptomatic participants. Duplicate records, abstracts and unpublished materials were removed. Full-text copies of the retrieved records were screened for the same criteria. If the full text copy was not retrievable through database or electronic search, the corresponding authors of the studies were contacted. If no response was received or the authors were not able to provide a copy of the selected manuscript, the article was excluded from the investigation. Reference lists of relevant publications were also screened.

### Selection of studies

**Fig. 1:**
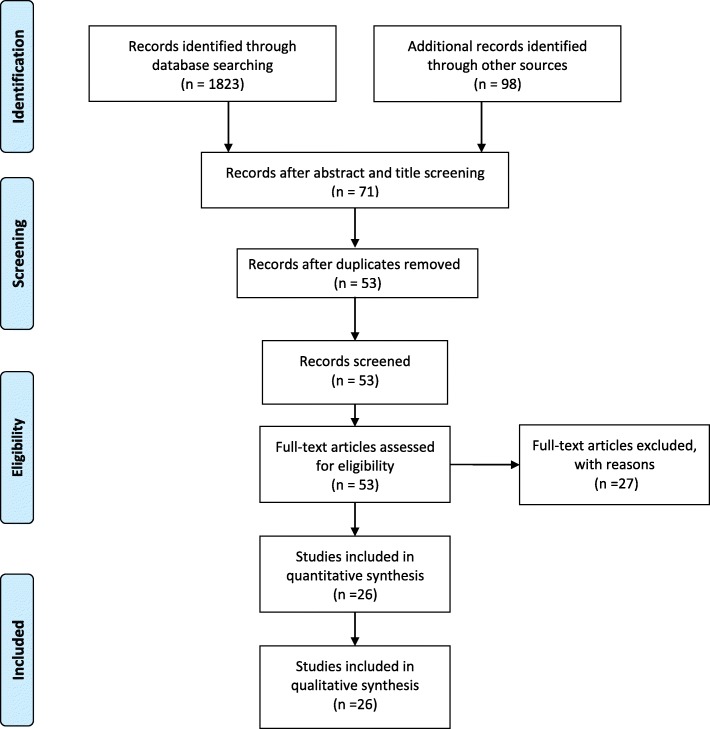
PRISMA flow diagram illustrating the different phases of study inclusion

Selection was conducted independently by two reviewers. Any disagreement was resolved through negotiation. All of the identified records from each database were combined into a single End Note file (End Note Version X7.5; Thompson Reuters, New York, USA) and subsequently screened for relevance using title and abstract. The full text of relevant studies was retrieved and assessed for eligibility against the inclusion criteria set above. The PRISMA flow diagram (Fig. 1) illustrates the process by which the manuscripts were selected and included in the final analysis.

### Quality assessment

The methodological quality of the randomized controlled trials was assessed using the PEDro scale. The PEDro scale is a 10-item tool designed to reliably assess the quality of physical therapy-based RCT’s, based on a 10-item checklist [31], where trials scoring at least 6/10 are deemed to be of ‘moderate to high quality’, although this cut-off point has yet to be validated [32]. Two authors independently assessed the PEDro score. Both authors successfully completed the PEDro consistency training. Data were entered and reviewed in Microsoft Excel spreadsheets (2016 Microsoft Corporation, Redmond, WA) and any disparity in scores resolved by discussion and thorough reevaluation.

### Statistical analysis

The studies included in the qualitative synthesis were classified according to the screened population (asymptomatic vs RCT’s). For each group, descriptive statistics were performed for the main variables and other presented indexes using STATISTICA for Windows (Statsoft, Inc., Ver. 10.0, Tulsa, OK, USA).

## Results

A total number of 1921 manuscripts were initially identified after the first search strategy in the four databases. After the application of the inclusion and exclusion criteria on each article’s title and abstract, 71 records were considered eligible, 18 records were removed as duplicates and 53 records were screened as full-text. Of the 53 records screened, 27 were removed due to non-MET studies, non-English manuscripts, missing data, not meeting the inclusion criteria or not being RCT’s. A final number of 26 original studies were included in the in the qualitative synthesis (Fig. 1 and Table 1).

**Table 1:** Summary of retrieved records included in the qualitative synthesis

Author	Sample size (n)	Population	Purpose	Protocol Duration	Anatomical administration of MET	Outcome	Pre-VAS	Post-VAS	Pre- ROM	Post-ROM	Other
Ballantyne et al. [22]	40	Asymptomatic participants	Flexibility increase	1 session	Hamstring	Increased ROM	n/a	n/a	167.3°	170.0°	n/a
Bindra [10]	30	Patients with chronic LBP	Effect of technique	6 sessions	Sacroiliac joint	Decreased Pain	6.5	1.6	n/a	n/a	Oswestry disability index
Burns et al. [33]	18	Asymptomatic participants	Flexibility increase	1 session	Cervical spine	Increased ROM	n/a	n/a	71.5°	+ 3.9°	n/a
Cassidy et al. [14]	100	Mechanical neck pain patients	Effect of technique	1 session	Upper trapezius	Pain decreased more than ROM improved	n/a	n/a	n/a	n/a	NRS-101 pain score (pre 37.7 vs post 20.4)
Fryer et al. [15]	52	Asymptomatic participants	Flexibility increase	1 session	Atlanto-axial joint	Increased ROM	n/a	n/a	52.41°	59.06°	n/a
Fryer et al. [34]	12	Asymptomatic participants	Neurophysiological responses	1 session	L5/S1 segment bilaterally	Decreased motor excitability (increased silent period and decreased H reflex)	n/a	n/a	n/a	n/a	n/a
Hamilton et al. [16]	90	Asymptomatic participants	Effect of technique	1 session	Sub-occipital region	No difference in pressure pain thresholds	n/a	n/a	n/a	n/a	n/a
Kamali et al. [35]	46	Women with thoracic Kyphosis	Effect of technique	15 sessions	Thoracic spine	Reduced Kyphosis and increased Strength	n/a	n/a	n/a	n/a	Sitting kyphosis angle pre 45.7° Sitting kyphosis angle post 40.6°
Küçükşen et al. [17]	82	Patients with chronic lateral epicondylitis	Effect of technique	8 sessions	Elbow	Improvement on strength, pain and function	7.39	3.28	n/a	n/a	DASH score pre 46.7 post 22.7
Laudner et al. [36]	39	Asymptomatic participants	Effect of technique	12 sessions	Pectoralis minor	Increased muscle length, Decreased forward scapular position	n/a	n/a	n/a	n/a	Pectorali s length pre 8.0 post 8.8, Scapular position pre 13.6 post 12.1
Lenehan et al. [21]	59	Asymptomatic participants	Increase trunk ROM	1 session	Thoracic spine	Increased ROM	n/a	n/a	26.4°	36.7°	n/a
Moore et al. [37]	61	Asymptomatic Athletes	Flexibility increase	1 session	Glenohumeral joint	Increased Joint mobility	n/a	n/a	−13.5	−8.5	n/a
Nagrale et al. [38]	60	Patients with non-specific neck pain	Effect of technique	12 sessions	Upper trapezius	Pain and Disability reduction	8.2	6.1	n/a	n/a	Neck disability index pre: 42.9 and post: 31.9
Oliveira-Campelo et al. [39]	117	Patients with unilateral latent trigger points	Effect of technique	1 session	Upper trapezius	Improves cervical motion and pain pressure sensitivity	n/a	n/a	57.4	58.7	n/a
Phadke et al. [13]	60	Neck pain population	Effect of technique	6 days	Upper trapezius and levator scapulae	Decreased VAS	5.5	1.64	n/a	n/a	n/a
Sadria et al. [12]	64	Patients with latent trigger points	Effect of technique	1 session	Upper trapezius	Decreased VAS and increased ROM	7.61	6.65	5.68 cm	6.56 cm	n/a
Sakshi et al. [40]	30	Patients with chronic neck pain	Effect of technique	8 sessions	Suboccipitalis, upper trapezius, and pectoralis major	Reduced Pain	6.4	1.6	n/a	n/a	Disability index pre 34.9 post 11.9
Schenk et al. [41]	18	Asymptomatic participants	Effect of technique	7 sessions	Cervical region	Increased ROM	n/a	n/a	Flexion: 51.8° Extention: 69.1° L Rotation: 35.6° R Rotation: 35.2°	Flexion: 54.4° Extention: 71.9° L Rotation: 40.8° R rotation: 43.4°	n/a
Schenk et al. [18]	26	Asymptomatic patients	Flexibility increase	8 sessions	Lumbar region	Increased ROM	n/a	n/a	13.8°	36.7°	n/a
Selkow et al. [7]	20	Patients with non-specific LBP	Effect of technique	1 session	Lumbar region	Decreased pain	2.9	2.5	n/a	n/a	n/a
Shadmehr et al. [20]	30	Female with Knee Rom impairment	Effect of technique	10 sessions	Hamstrings	Increased ROM	n/a	n/a	145.1°	167.2°	n/a
Smith et al. [19]	40	Asymptomatic participants	Flexibility increase	2 sessions	Hamstrings	Increased ROM	n/a	n/a	145.5°	154.0°	n/a
Tanwar et al. [42]	30	Patients with plantar fasciitis	Effect of technique	18 sessions	Gastrocnemius	Increased ROM and reduced pain	6.46	2.2	6.73	14.53	n/a
Ulger et al. [9]	113	Patients with chronic low back pain	Effect of manual therapy	18 sessions	Low back	Pain reduction and increase of functional parameters	6.9	2.08	n/a	n/a	n/a
Wilson et al. [8]	8	Patients with acute low back pain	Effect of technique	8 sessions	Low back	Change in ODI score (functional improvement)	n/a	n/a	n/a	n/a	Oswestry Disability Index pre 45 post 7
Yeganeh Lari et al. [11]	60	Female with latent trigger points	Effect of technique	4 sessions	Upper trapezius	Decreased VAS and increased ROM	6.8	4.6	23.8°	31.1°	n/a

In order to evaluate the different effects of MET, the screened populations were divided into asymptomatic subjects and symptomatic patients. There were 12 studies included in the asymptomatic population group [15, 16, 18–22, 33, 34, 36, 37, 41] (Table 2) comprising 485 participants and 14 studies included in the symptomatic population group [7–14, 17, 35, 38–40, 42] (Table 3) comprising a total of 954 patients, leading to a total of 1439 subjects analyzed.

**Table 2:** Summary of evidence of studies with asymptomatic participants

Authors	Sample size (n)	Sessions (n)	Anatomical administration of MET	Procedure	Outcome	Additional information	Comparison with other technique	
Ballantyne et al. [22]	40	1	Hamstring	5 s contraction at 75% of max – 3 s relaxation -at a point of discomfort – 4 contractions	Increased Rom	n/a	n/a	
Burns et al. [33]	18	1	Cervical spine	3 to 5 s contraction at 0.5 kg of pressure - 3 to 5 s relaxation – to a new barrier of motion – 2 to 4 contractions	Increased Rom with MET – Decreased with Sham	n/a	With Sham treatment (stretching for 3 to 5 s – return to neutral position - for 3 stretches)	
Fryer et al. [15]	52	1	Atlanto-axial joint	5 s: 5 s contraction at the first resistance point – 5 s relaxation – 3 times 20s: 20 s contraction at the first resistance point – 5 s relaxation – 3 contractions	Increased Rom in the MET groups. With the 5 s group showing the gretest increase.	The increase was greater in the direction of restriction compared to the direction of no restriction	Comparison of a 5 duration contraction with a 20 duration contraction + Sham therapy and control group.	
Fryer et al. [34]	12	1	L5/S1 segment bilaterally	5 s contraction at a first tissue tension point - relaxation – new barrier contractions – 3 contractions	Decreased H-reflex and silent period.	MET produces decreased motor excitability in the motor cortex and spinal cord.	Control Group	
Hamilton. et al. [16]	90	1	Subocciptal region	3 to 5 s contraction – 5 s relaxation – 3 contractions	Pressure pain thresholds increased in the MET compared to the sham but not HVLA procedure	n/a	Comparison with HVLA and Sham treatment	
Laudner et at. [36]	39	12	Pectoralis Minor	3 s stretch – 5 s contraction at 25% of max force – 4 contractions – no rest	Increased pectoralis length and decreased forward scapular position.	No increase of scapular upward rotation.	n/a	
Lenehan et al. [21]	59	1	Thoracic spine	5 s contraction at the first rotational barrier – no rest – new rotational barrier - four repetitions	Increased Trunk ROM	Restricted direction of treatment increased rotation more than non-restricted rotation	Comparison with control group.	
Moore et al. [37]	61	1	Glenohumeral Joint	5 s contraction at 25% max force - the participant then internally rotated the arm for 30-s and an active assisted stretch was applied - 3 contractions	Increased horizontal adduction and internal rotation ROM	n/a	Control group	
Schenk et al. [41]	18	7	Cervical region	5 s contraction – 3 s relaxation – increase of direction of limitation – 4 contractions	Increased Rom in all six ranges of motion of the cervical region.	n/a	Control group	
Schenk et al. [18]	26	8	Lumbar region	Greenman Protocol.	Increased extension of the Lumbar Spine	No increase of ROM in control group	Control group	
Shadmehr et al. [20]	30	10	Knee Rom	Hamstrings	10s contraction at 50% max force – 10s relaxation – greater resistance point – 3 contractions	Improvement of knee extension.	MET had an early effect on improving muscle’s flexibility compared with the passive stretch.	Passive stretch
Smith et al. [19]	40	2	Hamstrings	Chaitow MET: 7–10s contraction at 40% max force – 2-3 s relaxation – 30 s stretch to the palpated and/or tolerance to stretch – 3 contractions. Greenman MET: 7–10s contraction at 40% max force – 2-3 s relaxation – leg placed at a new barrier – 4 contractions.	Both Greenman and Chaitow approaches produced increases of active knee extension immediately after intervention.	No statistical differences between the two techniques.	Chaitow vs. Greenman protocol	
	*Total 485*	*Mean: 3.8*

**Table 3:** Summary of evidence of studies with Symptomatic patients

Authors	Sample size (n)	Sessions (n)	Patients symptom	Anatomical administration of MET	Procedure	Outcome	Additional information	Comparison with other technique
Bindra [10]	30	6	Chronic LBP	Sacroiliac Joint	Contraction for 8–10s at 25% max force – 2-3 s relaxation – 4-6 contractions – at the barrier of restriction	VAS and ROM improved at the end of treatment	MET and conventional therapy are both effective in managing lumbar back pain	Conventional therapy (ultrasound 5 mins, intensity of 1 W/cm2 and TENS for 10 mins 50–100 Hz)
Cassidy et al. [14]	100	1	Mechanical neck pain	Upper trapezius	5 s contraction – 4 contractions – at the restricted joint movement	Pain decreased and ROM increased	HVLA technique has larger benefits than MET after a single application	Comparison with HVLA
Kamali et al. [35]	46	15	Thoracic Kyphosis	Thoracic spine	5–7 s contraction at 25% of max force – 5 contractions – at the barrier of movement	Reduced Kyphosis increased ROM	Manual therapy is as effective as exercise therapy in reducing thoracic kyphosis	Exercise Therapy
Küçükşen et al. [17]	82	8	Chronic lateral epicondylitis	Elbow	5 s contraction at 75% max force – 5 s relaxation – 5 contractions – at the resistance point	Decreased VAS, Decreased DASH score (Disabilities of the Arm, Shoulder and Hand), increased PFGS (pain-free grip strength)	MET and CSI improved the strength, pain, and functional status of patients. CSI is a better option as a short term option. MET is superior as a long term option.	Corticosteroid Injections (CSI)
Nagrale et al. [38]	60	12	Non specific neck pain	Upper trapezius	7–10s contraction at 20% max force – Relaxation phase – 30s stretch after contraction to a new resistance point – 3-5 contractions.	VAS, Neck disability index (NDI) and ROM.	Significantly greater improvements in pain and neck disability and lateral cervical flexion ROM were detected in favor of the INIT group	Integrated neuromuscular inhibition technique (INIT)
Oliveira-Campelo et al. [39]	117	1	Unilateral latent trigger points	Upper trapezius	5 s contraction at 25% max force – 5 s relaxation – new end point – 3-5 contractions	ROM, and pain thresholds improved after a single session.	Ischemic compression resulted with a more stable improvement	Passive stretch, ischemic compression, placebo, control.
Phadke et al. [13]	60	6	Neck pain	Upper trapezius and levator scapulae	7–10s contraction at 20% max force − 20 s stretch beyond the resistance barrier − 5 contractions	Reduced pain (VAS) and reduced NDI	MET and stretching are both effective in relieving pain and reducing disability.	Static stretching
Sadria et al. [12]	64	1	Latent trigger points	Upper trapezius	7–10s contraction at 20% max force – relaxation phase – 30s stretch at the restriction barrier -	VAS reduction	Both techniques are equivalent for treating latent trigger points	Active release
Sakshi et al. [40]	30	8	Chronic neck pain	Suboccipitalis, Upper Trapezius and Pectoralis Major.	7–10s contraction with mild effort – new barrier – 3 contractions.	Reduction of neck disability index, Reduced forward head posture and pain	MET was superior to exercise intervention.	Exercise intervention
Selkow et al. [7]	20	1	Acute LBP	Lumbar region	5 s contraction – 5 s relaxation – 4 contractions	VAS decreased	n/a	Control group
Tanwar et al. [42]	30	18	Plantar fasciitis	Gastrocnemius	7–10s contraction at 20% max force – new restriction barrier – 30s stretch – 3 contractions	Increased ROM, improved foot functional index (FFI) and reduced pain	n/a	Static Stretch
Ulger et al. [9]	113	18	Chronic LBP	Low back	8 s contraction at 30% of max force – new stretch position - repeated until necessary	Pain severity reduction, Decreased Oswestry disability index (ODI), Improved quality of live levels	MET and spinal mobilization are equally effective on pain, function and quality of life. MET is more effective for pain during activity and functional parameters.	Spinal mobilization
Wilson et al. [8]	8	8	Acute LBP	Low back	5 s contraction – new barrier of motion – 4 contractions	Reduction in ODI score	MET elicited superior changes compared to control group.	Control
Yeganeh Lari et al. [11]	60	4	Latent trigger points	Upper Trapezius	7–10s contraction at 20% max force – relaxation phase – new barrier of motion – 30s stretch – 3-5 contractions	Reduced VAS, Increased neck ROM and increased pressure pain thresholds	MET+Dry needling was more effective in increasing rom and reducing pain than the 2 techniques alone.	Dry needling and MET+dry needling
	*Total: 954*	*Mean: 7.6*						

All reported data to follow are reported as means for each study. The 14 studies included in the symptomatic population were all RCT’s and these were subjected to PEDro quality assessment evaluation. Not all studies from the asymptomatic population group were RCT’s.

### Quality assessment of included RCT’s

The PEDro scores ranged from 2/10 to 9/10, with 10/14 RCT’s achieving ‘moderate to high quality’ scores (6/10). The overall PEDro risk of bias score of all the included studies was 6.4/10. The lowest scores for the included studies were achieved in item 6 (blinding of therapist) and item 7 (blinding of assessors) for scores of 1.4/10 and 2.9/10, respectively. A breakdown of PEDro scores for each trial is shown in Table 4.

**Table 4:** Quality assessment for included studies using the PEDro Score

Authors	1	2	3	4	5	6	7	8	9	10	11	Tot.
Bindra [10]	✓	✓		✓				✓		✓	✓	5
Cassidy et al. [14]	✓	✓		✓				✓	✓	✓	✓	6
Kamali et al. [35]	✓	✓	✓	✓				✓	✓	✓	✓	7
Küçükşen et al. [17]	✓	✓	✓	✓			✓	✓	✓	✓	✓	8
Nagrale et al. [38]	✓	✓	✓	✓	✓			✓	✓	✓	✓	8
Oliveira-Campelo et al. [39]	✓	✓	✓	✓			✓		✓	✓	✓	7
Phadke et al. [13]	✓	✓	✓	✓				✓	✓	✓	✓	7
Sadria et al. [12]	✓	✓	✓	✓	✓			✓	✓	✓	✓	8
Sakshi et al. [40]	✓	✓		✓						✓	✓	4
Selkow et al. [7]	✓	✓	✓	✓	✓	✓	✓	✓	✓			8
Tanwar et al. [42]	✓	✓		✓								2
Ulger et al. [9]	✓	✓	✓	✓	✓	✓	✓		✓	✓	✓	9
Wilson et al. [8]	✓	✓			✓				✓	✓		4
Yeganeh Lari et al. [11]	✓	✓		✓				✓	✓	✓	✓	6
Mean	10	10	5.7	9.3	3.6	1.4	2.9	6.4	7.9	8.6	7.9	6.4

### Asymptomatic population

Of the twelve included studies, ten investigated the effects of MET on joint ROM [15, 18–22, 33, 36, 37, 41], one the effect of MET on PPT [16] and one the effects of MET on corticospinal and spinal reflex excitability [34]. Three studies targeted the hamstring muscles [19, 20, 22], two studies the lumbar region [18, 34], one study the thoracic spine [21], one study the pectoralis minor [36], one study the glenohumeral joint [37] and four studies the cervical spine [15, 16, 33, 41]. The number of treatment sessions retrieved from the studies and included in this review ranged between one and twelve. Seven studies applied MET within a single session [15, 16, 21, 22, 33, 34, 37], one study provided two MET sessions [19], another provided seven MET sessions [41], only one study provided eight MET sessions [18], only one study provided ten MET sessions [20] and only one study provided twelve MET sessions [36].

All of the above protocols were applied on the restricted side of a targeted joint in the asymptomatic subjects. Different procedures for applying MET were presented by different authors. Ballantyne et al. [22] applied four contractions of 5 s each at 75% of the participant’s maximal force with a three-second relaxation phase between each contraction on the hamstring muscle. Similarly, Schenk et al. [18] implemented a non-specified force of contraction with a MET treatment applied for 4 weeks on the lumbar region (at the L5 and S1 intersegment junction). The results provided by Ballantyne et al. demonstrate an increase in hamstring passive extensibility, although such an increase was also exhibited in the control group. Schenk et al. [18] showed that MET were able to increase lumbar active extension compared to a control group. Burns et al. [33] applied two to four contractions of 5 s each with 0.5 kg of pressure and a three to 5 s relaxation phase between each contraction applied to the cervical spine. These results showed a significant difference between pre- and post-treatment and between MET and the control group for side-bending and rotation of the cervical spine. Fryer et al. [15] compared two different MET protocols for ROM increases: the first protocol applied three contractions of 5 s each, with a 5 s rest between each contraction and the second applied three contractions of 20 s each, with a 5 s rest between each contraction applied on the atlanto-axial joint. The results reveal that the 5 s contraction protocol increased active ROM on the restricted side of the atlanto-axial joint to a greater extent than the 20 s contraction protocol and the sham therapy used for comparisons (stretching). Similar protocols to the 5 s protocol of Fryer and colleagues [15] are those of Hamilton et al. [16] and Fryer et al. [34]. Hamilton and colleagues compared the MET technique to a high velocity low amplitude (HVLA) technique, a short, quick thrust over the restricted joints with the goal of restoring normal range of motion and a sham treatment to decrease sub-occipital PPT. All the recruited participants were free of: (1) neck pathologies, (2) long-term cortico-steroid use, (3) vertebro-basilar insufficiency (4) chronic pain or (5) headaches. A hand-held electronic algometer consisting of a pressure transducer applied a pressure to the suboccipital area (between C0 and C2) of 30 kPa/s. When the pressure changed into a sensation of pain, the participants pushed a button and stopped the algometer. Such experiment provided evidence that MET is more effective than the sham treatment but equal to the HVLA in reducing PPT.

Fryer et al. [34] investigated the effects of MET on corticospinal and spinal reflex excitability and a single application of MET to the lumbosacral joint produced a significant decrease in corticospinal and spinal reflex excitability, suggesting a decrease in motor excitability. The physiological mechanism was shown to act through an increase of the silent period of motor evoked potential and a reduction of the H-reflex amplitude. Both effects are associated with inhibition of the motor excitability of the motor cortex and the spinal cord.

Laudner et al. [36] applied MET using four contractions of 5 s’ duration, each at 25% of the patient’s maximal force, with a 3 s stretch directed to the pectoralis minor muscle. There was no relaxation phase between contractions. The results of Laudner et al.’s study showed an increase of the pectoralis minor length (length in cm/participant height in cm × 100: pre 8.0 ± 6 0.5 vs. post 8.8 ± 0.5, *p* < 0.001) after a six-week intervention and decreased forward scapular position when compared to a control group which did not receive any intervention (length in cm/participant height in cm × 100: pre 7.9 ± 0.5 vs. post 7.7 ± 0.5, *p* = .67). The scapular position was measured with the participant with the shoulders touching a wall. The perpendicular distance from the wall to the anterior portion of the acromion was the calculated forward scapular position. A similar protocol of Laudner et al. [36] was used by Lenehan et al. [21] and was able to achieve an increase in trunk rotation on the restricted side of rotation in the analyzed population.

Moore et al. [37] applied MET to the glenohumeral joint, for one group to the horizontal abductors and the second group to external rotators for three contractions of 5 s each at 25% of the maximal force of each patient. The horizontal abduction group was then asked to adduce the arm for 30 s after each contraction whereas the external rotator group was asked to actively rotate the arm internally for 30 s after each contraction. The results were then compared to those of a control group which did not receive any intervention. Dominant arm glenohumeral internal and external rotation ROM and glenohumeral horizontal adduction ware passively measured before the MET intervention. The results of Moore et al. [37] showed that MET applied to the horizontal abductors increased glenohumeral ROM internal rotation and adduction to a greater extent than MET applied to the external rotators. Both groups had significant increases of internal rotation and adduction when compared to the control group.

The study performed by Shadmehr et al. [20] applied a MET protocol in women on their hamstring muscles. Each participant prior to the application of the MET protocol was assessed for passive stretch of their hamstring muscles, in order to evaluate hamstring flexibility. The subsequently applied MET protocol consisted of three contractions of 10 s each at 50% of the patient’s maximal force with a ten-second relaxation phase between each contraction. The authors then compared the MET protocol to a static stretch performed for three sets. Each set was composed of three contractions of 10 s each. During each contraction the leg was held for each of the 10 s at the first resistance point of the knee joint perceived by the therapist. The authors reported no significant difference between MET and the static stretching in improving hamstring flexibility.

Two primary protocol types are implemented in the use of MET: the Greenman and Chaitow. The Greenman protocol consists of four contractions of seven to 10 s each performed at 40% of the patient’s maximal force with a three-second relaxation phase between contractions. The Chaitow Protocol consists of three contractions of seven to 10 s each performed at 40% of the patient’s maximal force with a three-second relaxation phase followed by a 30 s stretch applied at the palpated barrier of restriction. Schenk and colleagues [41] applied the Greenman protocol on the cervical region of the spine. The restriction point of the cervical region was found by the practitioner and if the subject had a limitation in extension, left rotation and left side bending the practitioner would passively introduce extension, left rotation and left side bending to the point of the restriction barrier. Each subject was then asked to produce a small isometric force away from the direction of restriction against the practitioner’s hand. The authors report that the application of the Greenman protocol for 4 weeks increased ROM of the cervical region in all six ranges of motion. Smith et al. [19] compared the Greenman and the Chaitow protocols described above, applied to subjects’ hamstring muscles to improve their extensibility. Each participant was measured for passive ROM of the hamstring muscles. The results obtained from the two protocols highlight that both are effective in increasing hamstring extensibility with no significant difference between the two groups. A summary of the MET procedures is shown in Table 5.

**Table 5:** Summary of the MET protocols applied in asymptomatic subjects

Author	Repetitions (n)	Contraction time (s)	Contraction force	Relaxation phase (s)	Stretch (s)
Ballantyne et al. [22]	4	5	75%	3	none
Burns et al. [33]	4	3	0.5 kg	3 to 5	none
Fryer et al. [15]	3	5	n/a	5	none
Fryer et al. [15]	3	20	n/a	5	none
Fryer et al. [34]	3	5	n/a	5	none
Hamilton et al. [16]	3	3 to 5	n/a	5	none
Laudner et at. [36]	4	5	25%	0	none
Lenehan et al. [21]	4	5	n/a	0	none
Moore et al. [37]	3	5	25%	n/a	30
Schenk et al. [41]	4	5	n/a	3	none
Schenk et al. [18]	4	7 to 10	40%	3	none
Shadmehr et al. [20]	3	10	50%	10	none
Smith et al. [19]	3	7 to 10	40%	3	30
Smith et al. [19]	4	7 to 10	40%	3	none
Median	3.5	5	40%	3	30

### Symptomatic population

The goal of the prescriptions for MET in the studies evaluated in this review were variable. Of the fourteen included studies, seven investigated the effects of MET on chronic pain [9, 10, 13, 14, 17, 40, 42], with two of these intended to treat chronic LBP [9, 10], three intended to treat chronic neck pain (CNP) [13, 14, 40], one plantar fasciitis [42] and one chronic lateral epicondylitis [17]. Two studies investigated the effects of MET on acute pain [7, 8]. Four studies investigated the effects of MET on trigger points [11, 12, 38, 39], all in the upper trapezius and one study examined the effects of MET on thoracic kyphosis [35].

The number of sessions for a typical MET prescription varied considerably. The range of those evaluated in this review is one to eighteen. Of the included studies, four applied MET within a single session [7, 12, 14, 39], one study provided four MET sessions [11], two studies provided six MET sessions [10, 13], three studies provided eight MET sessions [8, 17, 40], one study provided twelve MET sessions [38], one study provided fifteen MET sessions [35] and two studies provided eighteen MET sessions [9, 42].

#### MET and chronic pain

Two of the retrieved records regarding chronic pain investigated the effects of MET on LBP. The study by Bindra et al. [10] applied four to six contractions of eight to 10 s each at a force of 25% of the patient’s maximal force with a relaxation phase of two to 3 s on the sacroiliac joint in order to reduce chronic LBP. The patients were included in the study if they had tenderness over the sacroiliac joint, a mechanical LBP and hypomobility of the sacroiliac joint. The joint was treated for the appropriate dysfunction identified and for each patient, the restriction barrier was found in order to correctly apply the MET technique. The authors compared MET with conventional therapy (ultrasound for 5 min, intensity of 1 W/cm^2^ and TENS for 10 min 50–100 Hz), revealing that MET was the most beneficial treatment to manage pain and increase ROM. The Visual Analog Scale (VAS) used to assess pain decreased five points compared to baseline in the MET group and three points for the conventional therapy. The Oswestry Disability Index (ODI) also decreased 18% after six treatments in the MET group and 13% in the conventional therapy group. Ulger et al. [9] aimed to treat patients complaining of LBP. The authors applied an eight-second contraction at 30% of the patient’s maximal force until a relaxation of the targeted muscles was achieved. The MET procedure was applied on the quadratus lumborum and piriformis muscles. The treatment was applied for 18 sessions and compared to spinal mobilization. At the end of the treatment, MET was found more effective in reducing pain (VAS: pre 7 compared to post 2, *p* < 0.001) and the ODI (pre 46.4 compared to post 18.9, p < 0.001) compared to spinal mobilization (VAS: pre 5 compared to post 2, *p* = 0.979 and ODI pre 43.5 compared to post 23.5, *p* = 0.083).

Three of the retrieved records regarding chronic pain investigated the effects of MET on CNP. Sakshi and colleagues [40] investigated the effects of MET applied with three contractions of 7 to 10 s each at a mild effort in order to reduce pain, disability and forward head position in patients with CNP. The MET procedure was applied on the suboccipitalis, the upper trapezius and the pectoralis major muscles. After eight treatments, there was a reduction of the Neck Disability Index (NDI) (MET: pre 34.95 ± 9.74 compared to post 11.99 ± 4.42, *p* < 0.0001, exercise intervention: pre 34.50 ± 5.92 compared post 22.80 ± 6.79, *p* < 0.01), reported VAS pain (MET: pre 6 ± 1 compared post 2 ± 1, p < 0.0001; exercise intervention: pre 7 ± 1 compared to post 4 ± 1, p < 0.01), and forward head posture (MET: pre 51.70 ± 1.70% compared post 47.18 ± 1.63%, p < 0.01, exercise intervention: pre 52.96 ± 3.70% compared post 50.74 ± 3.91%, p < 0.01). The sample had been compared to an exercise intervention, which revealed a significant difference between the two groups in all measures (p < 0.01 for NDI and reported pain and *p* = 0.04 for forward head posture, which was measured sitting on a chair, calculating the distance from the chair backrest to the tip of the chin).

Phadke et al. [13] compared the effects of MET and static stretching on pain and functional disability. The protocol used by Phadke and colleagues involved three contractions of seven to 10 s each using 20% of maximal isometric contraction with a 20-s stretch between each contraction. The stretching protocol involved five repetitions of 20 s holds. The MET and stretching protocols ware applied to the upper trapezius and levator scapulae muscles. After six treatments, the VAS score and the NDI decreased significantly (VAS: pre 6 compared to post 2, *p* < 0.001 NDI: pre 17.25 compared to post 8.03, p < 0.001) compared to the stretching group (VAS: pre 5 compared to post 2, p < 0.001 NDI: pre 17.21 compared to post 9.6, p < 0.001) (Intragroup difference VAS *p* = 0.020 and NDI *p* = 0.024). Cassidy et al. [14] compared the effects of HVLA manipulation and MET manipulation in a sample of persons with CNP. CNP was assessed through the 101-point numerical rating scale. The results after treatment were in favor of the MET, in which a reduction of 17 points was achieved, while that the other treatment resulted in a reduction of 10.5 points in the 101-point numerical rating scale score. The protocol applied by Cassidy et al. [14] comprised four contractions of 5 s each. The authors, however, specify neither the duration of the relaxation phase nor the force applied to each contraction. The MET was applied on the muscle responsible for restricting joint movement.

One of the studies included in this review investigated the effects of MET in patients with a history of at least a month of plantar fasciitis and compared the effects with those from static stretching. The MET protocol used by Tanwar et al. [42] comprised three contractions of seven to 10 s each at 20% of the patient’s maximal force with a 3 s relaxation phase and a 30 s stretch after the relaxation phase between each contraction. The contractions were applied to the soleus and gastrocnemius muscles. The authors analyzed the ROM of the ankle, the foot functional index and pain through the numerical pain rating scale. ROM, the foot functional index and the numerical pain rating scale all improved to a significantly greater extent (*p* < 0.05) after the MET protocol (mean ROM: pre 6.7° compared to post 14.5° foot functional index: pre 43.9 compared to post 24.5 numerical pain rating scale: pre 6.5 compared to post 2.2) compared to the static stretching protocol (mean ROM: pre 6.4° compared to post 10.5° foot functional index: pre 43.6 compared to post 29.8 numerical pain rating scale: pre 6.3 compared to post 3.3). The protocol was applied for eighteen sessions over a period of 4 weeks.

Küçükşen et al. [17] analyzed the effects of eight MET treatments compared to a corticosteroid injection for chronic lateral epicondylitis. The MET protocol used by Küçükşen et al. comprised five contractions of 5 s each at 75% of the patient’s maximal force with a five-second relaxation phase between each contraction. Each contraction targeted the hand pronator muscles.

The study assessed pain-free grip strength, reported VAS values and the “Disabilities of the Arm, Shoulder and Hand questionnaire (DASH)”, respectively. The grip strength of the affected side was presented as a ratio of the maximum grip strength of the unaffected side. The measurements were performed at baseline, 6, 26, and 52 weeks after the treatments (baseline measurements of pain-free grip strength MET: 40.46 ± 17.26% compared to corticosteroid injection 44.00 ± 18.64%, *p* = .495, VAS MET: 7.39 ± 1.07 compared to corticosteroid injection 7.17 ± 1.07, *p* = .330, DASH MET: 46.73 ± 11.88 compared to corticosteroid injection 45.63 ± 10.40, *p* = .666). At the six-week evaluation, all measurements improved in both the corticosteroid injection group and the MET group, however the values for the corticosteroid group were significantly greater than those of the MET group (pain-free grip strength MET: 60.95 ± 19.07% compared to corticosteroid injection 72.4 ± 19.54%, *p* < 005, VAS MET: 4.38 ± 2.08 compared to corticosteroid injection 2.98 ± 2.49, *p* = .004, DASH MET: 26.25 ± 15.40 compared to corticosteroid injection 21.10 ± 14.02, *p* = .113). At 26 and 52 weeks, the MET group scored significantly better in all measurements. The corticosteroid group at 26 and 52 weeks had the tendency to relapse (26 weeks pain-free grip strength MET, 68.90 ± 19.15% compared to corticosteroid injection 61.45 ± 19.03%, *p* = .034, VAS MET, 4.00 ± 2.59 compared to corticosteroid injection 5.29 ± 2.04, *p* = .016, DASH MET: 23.78 ± 17.50 compared to corticosteroid injection 27.84 ± 14.91, *p* = .079 and 52 weeks pain-free grip strength MET: 75.08 ± 26.19% compared to corticosteroid injection 62.24 ± 21.83%, *p* = .007, VAS MET: 3.28 ± 2.86 compared to corticosteroid injection 4.95 ± 2.36, *p* = .001, DASH MET = 22.56 ± 20.29 compared to corticosteroid injection 27.03 ± 15.45, *p* = .061). No patients in the MET group reported side effects from the treatment, whereas three participants out of forty-one experienced side effects in the corticosteroid injection group.

#### MET and acute pain

Only two studies analyzed the effects of MET on acute pain. Wilson et al. [8] evaluated twelve patients with acute LBP. The authors applied a MET protocol consisting four contractions for 5 s each. Neither relaxation phase nor force applied were specified. The procedure was applied on each patients restricted side, directly targeting L3. The authors assessed the ODI before and after the application of eight treatments and compared the outcomes with a control group that underwent a manipulative sham treatment. The ODI measures were significantly improved (*p* < 0.05) in the MET group (pre 45 vs post 7 with a mean decrease of 83%) compared to the control group (pre 44 vs post 15, with a mean decrease of 65%). Another study by Selkow and colleagues [7] applied four contractions of 5 s each with a 5 s relaxation phase on the hamstrings and the iliopsoas muscle to treat non-specific LBP. VAS significantly decreased after a single application from 2.9 to 2.5 (*p* = .04) whereas it increased in the control group treated with a sham therapy from 1.4 to 3.5.

#### MET and myofascial trigger points

All of the studies analyzing MET and myofascial trigger points applied the techniques to the upper trapezius. All patients were tested for active trigger points, defined as a tender nodule in a taut band that referred pain beyond the area of contact. The patients were treated if suffering from non-specific neck pain, defined as non-articular or non-systemic or had painful symptoms in the upper trapezius.

Nagrale et al. [38] compared traditional MET to an integrated neuromuscular inhibition technique, a specific type of treatment for trigger points. Both treatments were applied for twelve sessions. The outcome measures were the VAS, the NDI and ROM of the neck. The MET involved three to five contractions for 7 to 10 s each at 20% of the patient’s maximal force with a 2 to 3 s relaxation phase and a 30 s stretch between each contraction. The stretch was performed taking the head and neck into increasing degrees of side bending, flexion and rotation to advance the stretch placed on the muscle. The integrated neuromuscular inhibition technique was a sequence of ischemic compressions over the trigger point followed by strain-counter-strain techniques until a position of ease was found. This procedure was repeated three to five times. Both groups revealed significant improvements in all the outcome measurements, although the improvements presented by the integrated neuromuscular inhibition technique group were significantly greater than those of the MET group.

A similar study design implemented by Oliveira-Campelo et al. [39] comprised three to five contractions of 5 s each at 25% of the patient’s maximal force with a five-second relaxation phase. The MET protocol was compared to an ischemic compression group, a passive stretching group, a placebo and a no-treatment group. ROM, VAS and pain pressure sensitivity were assessed at baseline, after 10 min, 24 h after the treatment and a week later. After a single treatment, pain thresholds and ROM of contralateral lateral flexion and ipsilateral rotation improved in both the manipulative treatment groups (MET: pain thresholds pre 1.8 ± 0.4 kg/cm2 compared to post 2.3 ± 0.4 2 kg/cm2, *p* < 0.01; mean ROM contralateral flexion: pre 39.8 ± 4.6° compared to post 45.2 ± 4.7°, p < 0.01; mean ROM ipsilateral rotation: pre 70.4 ± 5.7° compared to post 73.4 ± 5.1°, *p* < 0.01. Ischemic compression: pain thresholds pre 1.7 ± 0.3 kg/cm2 compared to post 2.9 ± 0.4 kg/cm2, p < 0.01; mean ROM contralateral flexion: pre 39.8 ± 5.1° compared to post 46.8 ± 5.4°, p < 0.01, mean ROM ipsilateral rotation: pre 71.2 ± 5.7° compared to post 76.5 ± 6.7°, p < 0.01). However, the improvements measured a week after the treatment seem to have been better maintained in the ischemic compression group compared to the MET group.

Sadria et al. [12] compared the effects of MET to a form of ischemic compression (active release). The authors measured VAS before and after a single application of the techniques. Active release consisted of a compression of the trigger point followed by an active motion of the patient’s neck from a shortened position to an elongated position involving a contralateral neck side flexion and ipsilateral neck rotation. The MET protocol consisted of four contractions ranging 7 to 10 s in duration at 20% of the patient’s maximal force with a three-second relaxation phase. During the relaxation phase, the head and the neck were eased into increasing degrees of side bending and rotation, this position being held for 30 s. The outcome of the study reports a reduction in the VAS in both groups with no significant difference between the two treatments.

The last included study in this review analyzed the effects of MET on latent trigger points [11] comparing manual treatment to dry needling. The outcome measures reported by the study were reported score on VAS, PPT and range of active contralateral flexion. The patients were divided into three groups: dry needling alone, MET alone and dry needling plus MET. MET was applied with three to five contractions of 7 to 10 s each at 20% of the patient’s maximal force with a two to 3 s relaxation phase and a 30-s stretch between each contraction. All groups significantly improved in ROM, VAS, and pressure pain threshold. However, the combination of MET and dry needling was more effective than either treatment alone.

#### MET and other dysfunctions

Of the retrieved records, only one study analyzed other types of dysfunctions than those described above. The study of Kamali et al. [35] applied a MET protocol consisting five repetitions of five to 7 s at 25% of the patient’s maximal force in order to treat postural hyperkyphosis. For each patient, the authors analyzed the dorsal tract of the vertebral column and identified which vertebrae presented the greatest movement restriction in extension. The therapist then placed his/her hand on the spinous process of the vertebra in order to move it to the end of the extension barrier and applied the MET protocol above described. In addition to applying MET, the therapist applied a massage to the back extensor muscles for 10 min, a mobilization of the thoracic spine and a myofascial release technique. The exercise therapy comprised a combination of strengthening and stretching exercises. Both treatments were carried out for a five-week period. The outcome measures were thoracic kyphosis angle measured by a six-camera motion analysis system and muscle strength of the back extensor muscles measured through a dynamometer. All measures improved post-treatment in both groups with no significant differences between groups (Kyphosis angle in upright sitting: MET increase from baseline 2.51 ± 1.92°; Exercise intervention increase from baseline 3.17 ± 2.35°, *p* = 0.855. Kyphosis angle in relaxed sitting: MET increase from baseline 5.16 ± 3.90°; Exercise intervention increase from baseline 5.18 ± 4.25°, *p* = 0.935. Muscle strength: MET increase from baseline 26.76 ± 22.65 N; Exercise intervention increase from baseline 27.28 ± 16.50 N, *p* = 0.175).

A summary of the MET procedures is shown in Table 6.

**Table 6:** Summary of the MET protocols applied in symptomatic patients

Author	Repetitions (n)	Contraction time (s)	Contraction force	Relaxation phase (s)	Stretch (s)
Bindra [10]	4 to 6	8 to 10	25%	2–3	none
Cassidy et al. [14]	4	5	n/a	n/a	n/a
Kamali et al. [35]	5	5 to 7	25%	0	none
Küçükşen et al. [17]	5	5	75%	5	none
Nagrale et al. [38]	3 to 5	7 to 10	20%	2–3	30
Oliveira-Campelo et al. [39]	3 to 5	5	25%	5	none
Phadke et al. [13]	5	7 to 10	20%	n/a	20
Sadria et al. [12]	4	7 to 10	20%	3	30
Sakshi et al. [40]	3	7 to 10	Mild effort	n/a	none
Selkow et al. [7]	4	5	n/a	5	none
Tanwar et al. [42]	3	7 to 10	20%	3	30
Ulger et al. [9]	Until necessary	8	30%	n/a	none
Wilson et al. [8]	4	5	n/a	n/a	none
Yeganeh Lari et al. [11]	3 to 5	7 to 10	20%	2–3	30
Median	4	8	20%	3	30

## Discussion

The aim of this review was to understand the efficacy of MET on pain and joint range of motion, and to understand the differences between the different MET protocols in symptomatic and asymptomatic subjects. The quality assessment showed a “moderate to high” quality level of the included RCT’s.

The analyzed protocols for the asymptomatic subjects, comprised three or four contractions (mode: 4 contractions; median: 3.5 contractions) ranging from three to 10 s in duration (mode and median: 5 s) with contraction forces ranging from 25 to 75% of the patient’s maximal force (mode and median: 40%), a relaxation phase ranged between 0 and 5 seconds (mode and median: 3 s) and the stretch phase, which was not applied in 10 of the 14 protocols.

The only protocol directly comparing MET with and without a stretch was that of Smith et al. [19], in which the authors concluded that altering the duration of the stretch does not increase the effects of the technique on muscle extensibility. Fryer et al. [15] also compared the effect of a five- and twenty-second contraction time protocol and concluded that the five-second protocol was more effective in reducing the rotational asymmetry of the atlanto-axial joint. Therefore a shorter protocol can be suggested in an asymptomatic population if the aim of the MET is to increase joint ROM.

In symptomatic patients, the protocols comprised three to six contractions (mode and median: 4 contractions) extending in the range of 5 to 10 s, (mode and median: 8 s) with contraction forces ranging from 20 to 75% of the patient’s maximal force (mode and median: 20%), a relaxation phase between 0 and 10 s (mode and median: 3 s) and a stretch phase that was not present in 9 of 14 protocols. When reported, the stretch phase ranged between 20 to 30 s (mode and median: 30 s).

The range of contraction forces for MET protocols suggested by Chaitow [5] ranges between 15 and 40% of a person’s maximal contraction. The first case is usually applied in acute dysfunctions, whereas the second for chronic dysfunctions. In this systematic review the upper range of contraction forces used is 75% of a person’s maximal contraction, which is far from the suggested range of Chaitow. Only two studies however apply such high contraction intensity, Ballantyne et al. [22] in an asymptomatic population and Küçükşen et al. [17] for chronic lateral epicondylitis. The aim of Ballantyne was to acutely increase hamstring extensibility and such result was achieved by the authors, who attribute the immediate increase in ROM to an increased stretch tolerance. This could mean that a high intensity contraction could produce postsynaptic inhibitory mechanisms, resulting in lower excitation of the cortical and α-motor neurons, thereby modulating stretch perception [4]. It is unclear why Küçükşen et al. [17] performed a 75% contraction on a symptomatic population, however the results provided by the authors highlight that also with a higher contraction intensity it is possible to achieve positive clinical outcomes.

The results of the studies assessing the effects of MET on chronic LBP all showed decreases of the pain and disability indexes (VAS and ODI). In particular, Bindra et al. [10] compared the effects of MET to conventional treatment and both treatments were similarly effective in reducing LBP. Other reviews analyzing the effects of MET on LBP [43, 44] concluded that MET are moderately effective for chronic and non-specific LBP for managing pain and disability. There is no evidence that MET are ineffective for patients presenting with LBP. However, both reviews [43, 44] posit the necessity of producing higher methodological quality studies in the field.

Only two studies analyzed acute LBP [7, 8] having as total sample a number of 28 patients. Notwithstanding the limited retrieved records, both studies showed that MET was able to decrease pain and disability indexes after the treatment procedure. The targeted anatomical regions were the hamstrings, the iliopsoas and L3.

In regards to CNP, MET were compared to an exercise intervention, a stretching intervention and a mobilization intervention. In all three studies pertaining to CNP, pain and disability indexes were analyzed and showed that MET were the superior treatment compared to the other interventions for reduction in pain and disability. The study intervention periods range between 2 and 8 treatments.

The only study evaluating plantar fasciitis advocated an increase in ROM in parallel with decreased pain scores after the manipulative treatment [42]. Unfortunately, the study of Tanwar et al. [42] is of low methodological quality as reported in the PEDro scale, having no form of blinding, inadequate follow-up, no comparisons between groups and no measures of variability. Therefore, future research on the topic of plantar fasciitis is encouraged in order to evaluate the effects of MET.

Küçükşen and colleagues analyzed the effects of MET and corticosteroid injection after six, 26 and 52 weeks. Interestingly, the early phase of the treatment following the injection of corticosteroids was more beneficial in reducing pain and increasing pain free grip strength. However, the authors demonstrated a relapse after 26 and 52 weeks in the injection group, whereas a continuous reduction in elbow pain was shown in the MET group from 6 to 52 weeks.

Four studies evaluated the effects of MET on myofascial trigger points [11, 12, 38, 39] and successfully provided evidence that pain and disability indexes are reduced after the application of MET. However, other treatments such as ischemic compressions, integrated neuromuscular inhibition technique, Active Release technique and dry needling are equally (active releases and dry needling) or even more effective (ischemic compression and integrated neuromuscular inhibition technique) in reducing the negative symptoms of myofascial trigger points. Thus, in comparison to MET, more specific techniques are more appropriate in the treatment of myofascial trigger points.

### Limitations

There was a large heterogeneity in the MET protocols utilized. Of the 26 included studies, only 15 provided a full description of the treatment protocol (number of contractions, contraction time and force, relaxation phase if used and stretch duration, magnitude and hold time utilized between the contractions). Therefore, it is difficult to generally state which protocol is the most beneficial.

Future studies evaluating MET effectiveness are encouraged in order to identify which procedure may be more beneficial when treating different musculoskeletal disorders.

## Conclusions

MET are effective in improving reported pain, disability and joint range of motion in both asymptomatic subjects and symptomatic patients. The studies evaluated in this review have provided evidence that MET are specifically effective for alleviating chronic pain of the lower back and neck and chronic lateral epicondylitis. There is also evidence supporting MET as a beneficial therapy for reducing acute lower back pain and improving the related disability indexes. However, further evidence is needed to confirm MET as an effective treatment for plantar fasciitis and other musculoskeletal disorders. A definitive protocol for MET application, due to the heterogeneity of the results, could not be identified, and a future evaluation of the parameters of MET prescription is suggested.

## Abbreviations

CNP: Chronic Neck Pain
DASH: Disabilities of the Arm, Shoulder and Hand questionnaire
HVLA: High Velocity Low Amplitude
LBP: Low Back Pain
MET: Muscle Energy Techniques
NDI: Neck Disability Index ODI - Oswestry Disability Index
PNF: Proprioceptive Neuromuscular Facilitation
PPT: Pressure Pain Thresholds
RCT’s: Randomized Control Trials
ROM: Range Of Motion
VAS: Visual Analog Scale
